# Supplementary figures and images for: A fresh look at Cladarosymblema narrienense, a tetrapodomorph fish (Sarcopterygii: Megalichthyidae) from the Carboniferous of Australia, illuminated via X-ray tomography
Source: PeerJ. 2021 Dec 10;9:e12597. doi: 10.7717/peerj.12597 (PMC8667741; doi:10.7717/peerj.12597)

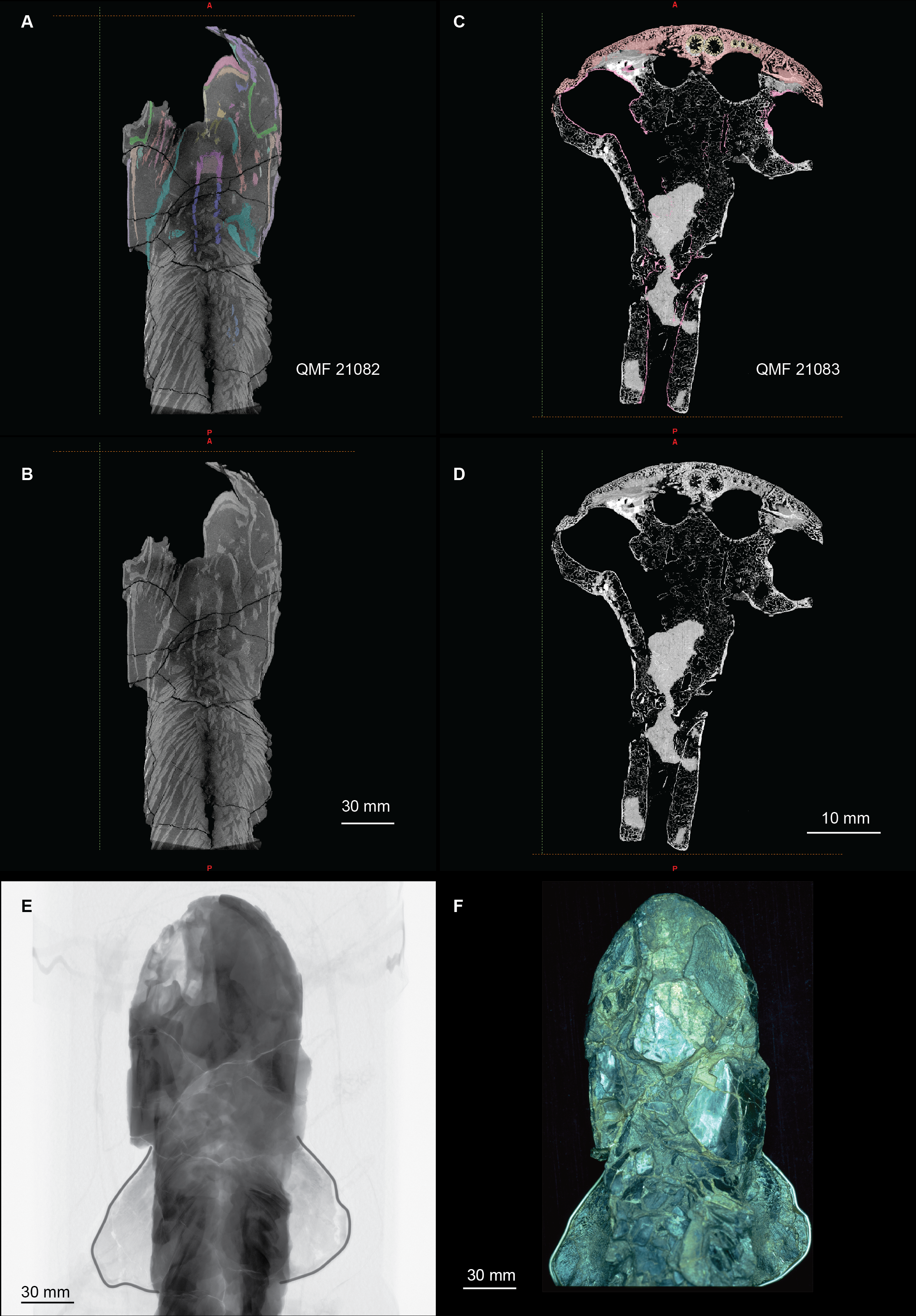

Supplement: Supplemental Information 1 [file peerj-09-12597-s001.png]
